# Change in Neighborhood Socioeconomic Status and Adherence to the Cancer Prevention Lifestyle Guidelines in Hispanic/Latino Adults: Results from the HCHS/SOL Study

**DOI:** 10.1158/2767-9764.CRC-23-0187

**Published:** 2023-10-02

**Authors:** Margaret S. Pichardo, Catherine M. Pichardo, Gregory A. Talavera, Linda C. Gallo, Charlene C. Kuo, Sheila F. Castañeda, Earle C. Chambers, Martha L. Daviglus, Amber Pirzada, Krista M. Perreira, Daniela Sotres-Alvarez, Tania Yadhira Peña Ortiz, Jesse J. Plascak

**Affiliations:** 1 Department of Surgery, Hospital of the University of Pennsylvania, University of Pennsylvania Health System, Philadelphia, Pennsylvania.; 2 South Bay Latino Research Center, Department of Psychology, San Diego State University, San Diego, California.; 3 Department of Psychology, San Diego State University, San Diego, California.; 4 Department of Behavioral and Community Health, University of Maryland School of Public Health, College Park, Maryland.; 5 Department of Family and Social Medicine, Albert Einstein College of Medicine, The Bronx, New York.; 6 Institute for Minority Health Research, University of Illinois Chicago, Chicago, Illinois.; 7 Department of Social Medicine, University of North Carolina School of Medicine, Chapel Hill, North Carolina.; 8 Department of Biostatistics, University of North Carolina School of Medicine, Chapel Hill, North Carolina.; 9 Weill Cornell Medical College, Weill Cornel Medicine, New York City, New York.; 10 Division of Cancer Prevention and Control, Ohio State University College of Medicine, Columbus, Ohio.

## Abstract

Neighborhood conditions are dynamic; the association of changing neighborhood socioeconomic factors with cancer preventive behaviors remains unclear. We examined associations of neighborhood socioeconomic deprivation, gentrification, and change in income inequality with adherence to the American Cancer Society Guidelines on Nutrition and Physical Activity for Cancer Prevention in The Hispanic Community Health Study/Study of Latinos (HCHS/SOL). The HCHS/SOL enrolled 16,415 adults, ages 18–74 years, at baseline (2008–2011), from communities in the Bronx, NY, Chicago, IL, Miami, FL, and San Diego, CA. Geocoded baseline addresses were linked to the 2000 decennial Census and 5-year American Community Survey (2005–2009 and 2012–2016) tracts to operationalize neighborhood deprivation index (NDI), gentrification, and income inequality. Complex survey multinominal logistic regression models estimated the relative risk ratio (RRR) with overall guideline adherence level (low, moderate, high) and by components—diet, physical activity, body mass index (BMI), and alcohol intake. Overall, 14%, 60%, and 26% of the population had low, moderate, and high ACS guideline adherence, respectively. NDI was negatively associated with risk of high (vs. low) guideline adherence [RRR = 0.87, 95% confidence interval (CI) = 0.78–0.98], although attenuated after controlling for individual socioeconomic status (SES; RRR = 0.89, 95% CI = 0.80–1.00), and associated with lower adherence to BMI recommendations (low vs. moderate RRR = 0.90, 95% CI = 0.84–0.97; high RRR = 0.86, 95% CI = 0.77–0.97). Gentrification was associated with higher likelihood of meeting the dietary recommendations (low vs. moderate RRR = 1.04, 95% CI = 1.01–1.07), but not with overall adherence or individual components. Change in income inequality was not associated with outcomes. Neighborhood deprivation may be negatively associated with ACS guideline adherence among Hispanic/Latino adults.

**Significance:**

This study provides new evidence on the link between neighborhood gentrification, changing income inequality and adoption and maintenance of cancer preventive behaviors in an understudied population in cancer research. We observed that while neighborhood deprivation may deter from healthy lifestyle behaviors, positive changes in neighborhood SES via the process of gentrification, may not influence lifestyle guideline adherence among Hispanic/Latino adults.

## Introduction

Adherence to the American Cancer Society Guidelines on Nutrition and Physical Activity for Cancer Prevention (1, 2), has been linked to lower obesity-related cancer risk (3–5). Obesity, a disease with a high burden among U.S. Hispanic/Latino adults (6), can be modified through healthful lifestyles to decrease obesity-related cancer risk (4, 5) and mortality (5) among Hispanic/Latino adults. Yet, adherence to these recommendations remains low among Hispanic/Latino adults (3–5). The high prevalence of obesity and adverse lifestyle behaviors in Hispanic/Latino individuals may be linked to sociocultural, economic, environmental, and structural factors that predispose individuals to poor health behaviors and risk of obesity-related cancers (7, 8).

The U.S. legacy of overt (e.g., slavery and segregation) and covert (e.g., residential, commercial, and educational redlining) racism led to the concentration of racially and ethnically minoritized communities in under-resourced neighborhoods (9, 10), thereby concentrating poverty (11, 12) and unequal exposures to environmental hazards (13, 14). For example, neighborhoods with high Hispanic/Latino segregation often have high levels of deprivation (12, 15) and are known to predispose residents to higher risk of obesity (16, 17) through fewer resources and opportunities to engage in healthy lifestyles and as such, may be less able to meet the guidelines for cancer prevention (18–20). These neighborhoods often lack infrastructure for physical and recreational activities, have low walkability, are unsafe/have more crime (21–25), and lack access to healthy and high-quality foods (24, 26) including fresh produce (27). Neighborhoods with high concentrations of low socioeconomic and racially and ethnically minoritized groups are more likely to have adverse and low quality food environments (28, 29), spaces with reduced walkability (24), limited recreational resources (25, 30), increased risk of obesity (31), and poor perceived health (32). In turn, these environments are known risk factors for poor diet quality (32–35) and physical inactivity (36). We previously linked residential areas with lower economic and racial privilege [i.e., racialized economic segregation (37)] and neighborhoods with high Hispanic/Latino segregation to low adherence to the overall ACS guidelines as well as lower likelihood of meeting the body mass index (BMI), physical activity, and alcohol recommendations (3).

The role of positive changes in neighborhood-level socioeconomic status (SES)—through the process of gentrification—on the health of residents remains unclear, with a growing body of literature presenting mixed or null results (38–40). According to Smith (41), gentrification is defined as “the process by which central urban neighborhoods that have undergone disinvestments and economic decline experience a reversal, reinvestment, and the in-migration of a well-off middle and upper-middle-class population.” On one end, gentrification may promote healthy lifestyles by increasing access to community resources such as parks, recreational areas, and healthy food options (42, 43). On the other end, gentrification may produce chronic stress by altering social networks and community cohesion (44, 45) and lead to voluntary or involuntary displacement of some residents due to increases in housing values and costs (46, 47). However, in a recent study of U.S. Hispanic/Latino adults, we reported no association between gentrification and change in income inequality with 6-year incidence of metabolic syndrome (39). Other studies on gentrification and health have found similar null or mixed associations [e.g., self-rated health (48), physical or mental health (49), incidence of hypertension (50), and mortality (51) among minoritized residents]. Gentrification may also influence income inequality and income polarization within communities that are undergoing an influx of new residents or displacement of long-standing residents (52, 53).

The intersection of low SES with residence in neighborhoods with rapidly changing income characteristics that make healthy lifestyles prohibitive despite being accessible, may amplify the risk of overweight and obesity. This in turn, may predispose Hispanic/Latino adults to developing obesity-related cancers and exacerbate existing cancer inequities (54, 55). To better understand how changing neighborhood economic environment influence lifestyle behavior change among U.S. Hispanic/Latino adults, our study aims to examine the associations between neighborhood deprivation, neighborhood gentrification, and neighborhood change in income inequality with adherence to the 2012 ACS Guidelines on Nutrition and Physical Activity for Cancer Prevention (1).

## Materials and Methods

### Study Population

The Hispanic Community Health Study/Study of Latinos (HCHS/SOL) is a longitudinal multicenter, community-based cohort study that aims to characterize the prevalence and incidence of disease burden in diverse Hispanic/Latino adults in the United States (56, 57). Between 2008 and 2011, 16,415 noninstitutionalized Hispanic/Latino adults (ages 18–74 at baseline) were recruited and enrolled across four sites: Miami, FL; San Diego, CA; Chicago, IL; and the Bronx County, NY from areas with high concentrations of Hispanic/Latino residents and communities with low residential mobility to maximize retention rates in follow-up visits. Participants self-identified as Cuban (*n* = 2,348), Dominican (*n* = 1,473), Mexican (*n* = 6,472), Puerto Rican (*n* = 2,728), Central American, (*n* = 1,732), and South American (*n* = 1,702). Briefly, the sampling strategy included a stratified two-stage area probability sampling of census block groups and households across four U.S. cities. Additional details on the study sampling have been published (56, 57). At baseline, a battery of questionnaires was administered to assess demographic and lifestyle factors, and anthropometric measurements were obtained. This study used participant data at baseline. The study protocol was approved by all participating Institutional Review Boards, and the research was conducted in accordance with the ethical principles of the Declaration of Helsinki. Study participants provided written informed consent.

Baseline participant addresses were geocoded and linked to 2005–2009, 2006–2010, and 2012–2016 5-year Census tract estimates of the American Community Survey using the IPUMS National Historical Geographic Information System (58) and the National Neighborhood Change Database produced by Geolytics (59) and the 2000 decennial census using tracts.

### Neighborhood SES

#### Neighborhood Deprivation Index

This measure was operationalized using 2010 Census tracts by an approach developed by Messer (60) and described in detail for this cohort previously (3, 61). Briefly, we employed a principal component analysis to identify a score based on the shared variance of four variables representing neighborhood-level SES: (i) percent of residents with less than a high school diploma, (ii) percent of residents with household incomes below 100% of the federal poverty level, (iii) percent of residents who are unemployed, and (iv) median household income. The score was standardized to a mean of 0 and SD of 1. Interpretation is based on 1-SD changed; higher values indicate more deprivation.

#### Neighborhood Change in Income Inequality

The Gini coefficient of income distribution was downloaded from the IPUMS National Historical Geographic Information System (58), and based on American Community Survey 2005–09 and 2012–2016 datasets. The approach employed by IPUMS to calculate this measure was developed and described in detail by Shrider and colleagues (62). This index reflects how similar household income are across households within a given census tract, with values ranging from 0 (perfect equality) to 100 (perfect inequality; ref. 63). We estimated percept change in neighborhood income inequality using the following formula: [(Gini_2012–16_ − Gini_2005–09_)/Gini_2005–09_ * 100], as previously done by others (64). A negative value in the change score indicates an improvement in income inequality, zero reflects no change, and a positive value reflects worsening income inequality over time. For this study, we scaled the coefficient to 10, thus, 1-unit change represents a 10% increase in inequality.

#### Neighborhood Gentrification

Following prior studies, we used data from the 2000 decennial census and the 2006–10 American Community Survey to calculate an index of gentrification based on percent changes of: college or more educated adults ages 25 or more, number of residents living below the federal poverty line, and median household income (39, 51, 65). Higher values indicate greater gentrification. Interpretation is based on a 1-unit change, with higher values indicating greater gentrification.

#### Neighborhood Immigrant Composition

Using the 2006–2010 American Community Survey data, we calculated percent of foreign-born residents in each census tract, with higher values representing a greater percent of foreign-born residents in a tract.

### American Cancer Society Guideline Adherence Score

For comparability with prior studies on American Cancer Society guideline adherence and cancer risk and outcomes among Hispanic/Latino adults (3–5), we operationalized the 2012 American Cancer Society Guidelines on Nutrition and Physical Activity for Cancer Prevention (1), rather than the updated version on June 2022 (2) using baseline participant data.

### Diet

Diet data came from two 24-hour dietary recall interviews (66) that assessed intake of specific foods, including traditional and cultural foods (67). The diet components were scored according to the following cutoffs: (i) fruits and vegetables—1 point for consuming ≥5 servings/day and 0 otherwise; (ii) total carotenoids—0, 1, or 2 points for being in the first, second, or third tertile of carotenoid intake; (iii) red and processed meat—divided into quartiles and assigned scores of 0–3 (lowest quartile = 3); and (iv) whole grains, defined as percentage of whole grains consumed (whole grains/total grains) then divided into quartiles and assigned a score of 0–3 (lowest quartile = 0). A final diet score was obtained by summing across the four diet components that ranged from 0 to 9. Dietary adherence was then classified as low (0–2 diet points), moderate (3–6 diet points), and high (7–9 diet points) adherence. Of note, our interpretation of the American Cancer Society dietary guidelines reflects the approach used in prior studies of cancer outcomes in Hispanic/Latino adults (4, 5, 68). Data-driven interpretation of the guidelines was used when a recommendation did not call for a specific intake quantity.

### Alcohol

Alcohol intake as grams per day was obtained from the dietary recall and was scored separately from the diet score. One drink was defined as 14 g of pure alcohol (69). The alcohol recommendation was operationalized as 2 points for nondrinkers (high adherence) and 1 point for consuming up to 1 or 2 drinks per day for women and men (moderate adherence), respectively, and 0 points if exceeding the alcohol recommendations (low adherence).

### Physical Activity

Objective moderate to vigorous physical activity (MVPA) was captured using accelerometry-measured data derived from accelerometer that participants wore for 7 days to assess frequency, duration, and intensity of their activity during that period. A detailed description of the accelerometer data has been published previously (70). The MVPA recommendation was operationalized as 2 points for engaging in ≥150 minutes/week of moderate or ≥75 minutes/week of vigorous activity per week (high adherence), 1 point for MVPA below recommended levels (moderate adherence), 0 points for 0 MVPA (low adherence).

### BMI

Anthropometric measures (height and weight) were collected during in-person visits to the study site including height to the nearest centimeter and weight to the nearest 0.1 kg. Self-reported weight and height at age 21 years were also collected at baseline and used when data were available. BMI was calculated using the formula kg/m^2^ and traditional cut-off points were used to indicate normal weight (BMI 18.5 to < 25.0 kg/m^2^), overweight (25.0 to <30.0 kg/m^2^) and obesity (≥30.0–50 kg/m^2^). The BMI recommendation was operationalized as 2 points for maintaining a BMI <25 kg/m^2^ at age 21 and at study entry (high adherence), 1 point for maintaining a BMI between 25 and 30 kg/m^2^ at either time (moderate adherence), and 0 points for BMI ≥30 kg/m^2^ at either point (low adherence).

### Composite Score

A composite guideline adherence score was derived from categorizations of dietary and alcohol intake, MVPA, and BMI components summed with possible range of “0” (does not meet recommendations) to “8” (meets all recommendations), and further categorized as low (0–3), moderate (4–5), and high (6–8) adherence.

### Covariates

We chose *a priori* confounders based on prior neighborhood studies in this population (3, 61), including health insurance status (yes, no), age categories (18–44, 45–65, >65), education (<high school, high school, some college, ≥college), household income (<$30,000, ≥$30,000, not reported), employment status (employed, unemployed), marital status (married/partnered, not married/partnered), smoking status (current, former, never), acculturation proxies [language preference (Spanish, English)], birthplace and duration of residence in the United States 50 states/DC (U.S. born, foreign/U.S. territory born <10 years, foreign/U.S. territory born ≥10 years), and Hispanic/Latino heritage. Neighborhood immigrant composition was defined as a neighborhood-level covariate.

### Statistical Analysis

All analyses accounted for HCHS/SOL complex survey sampling including stratification, clustering and sampling weights according to the guidelines established by the HCHS/SOL Coordinating Center. Separate sampling weights were used for analysis using accelerometry-measured variables. Of the 16,415 HCHS/SOL participants, 92.3% (*N* = 15,153) returned the accelerometer, and 77.7% (*N* = 12,750) provided at least 3 adherent days with 10 or more hours of wear time (70). Because of this large amount of missing accelerometry-measured outcomes (*n* = 3,933), analyses in this study were adjusted for missing data using inverse probability weighting (71, 72). We excluded participants with missing data on variables of interest (not mutually exclusive): home addresses (*n* = 316); residing outside of counties of interest (*n* = 370); BMI at study entry (*n* = 428); and intake of meat (*n* = 1,086), grains (*n* = 1,086), fruits (*n* = 1,086), vegetables (*n* = 1,086), nuts and legumes (*n* = 223), and carotenoids (*n* = 434). A missing indicator was used for sociodemographic factors, yielding an analytic sample of 11,909.

Weighted descriptive characteristics are provided for the total population. We calculated mean neighborhood scores by guideline adherence levels. We used multinominal logistic regression models to estimate the relative risk ratio (RRR) and 95% confidence interval (CI) of adherence to overall American Cancer Society guidelines by neighborhood measures. Given the clinical effects of smoking and smoking cessation on body weight (73), associations were also examined in a sample restricted to never smokers. In exploratory analysis, we also modeled individual guideline components to investigate whether overall guideline adherence driven by individual components. The proportional odds/parallel lines assumptions were examined using Akaike information criterion (AIC) and Bayesian information criterion (BIC) indices (74). Given that AIC and BIC were inconsistent, we also estimated ordered logistic regression models (OR and 95% CI estimates) and provide them as Supplementary Tables. Correlations between neighborhood indices are also provided in the Supplementary Tables.

We built three models to investigate the roles of potential confounders. In model 1, we adjusted for individual-level characteristics including, age, sex, marital status, acculturation, and insurance status. Model 2 adjusted for individual-level education and household income. Because some neighborhood risk factors may be confounders for other exposures, model 3 for the Gini income inequality index also added the neighborhood deprivation index and neighborhood immigrant composition, while models of gentrification additionally added neighborhood immigrant composition. All analyses were conducted using STATA 16 (75) and considered statistically significant at *P* < 0.05.

### Data Availability Statement

Data are maintained by the Hispanic Health Community Study/Study of Latinos and are available upon submitting a proposal to be approved by the publications committee. For more information, visit https://sites.cscc.unc.edu/hchs/. Data can also be accessed at National Heart Lung and Blood Institute (NHLBI) BioLINCC and in Database of Genotype and Phenotype (dbGaP), NIH maintained database of datasets and was developed to archive and distribute the results of studies that have investigated the interaction of genotype and phenotype. https://www.ncbi.nlm.nih.gov/gap/

## Results

In this population, the majority were ages 18–44 (57%), female (52%), not married (75%), had less than a high school education (33%), had incomes less than $30,000 (61%), were insured (50%), were foreign born and residing in the United States <10 years (51%), and had a Spanish language preference (76%; Table 1). Overall, 14%, 60%, and 26% of adults were classified as being low, moderately, or highly adherent, respectively, to the guidelines for cancer prevention.

**TABLE 1:** Weighted baseline characteristics of U.S. Hispanic/Latino adults, recruited from 2008–2011, *N* = 11,909

Participant characteristics	No. of participants	Weighted %
*Age, y*		
18–44	4,530	57.0
45–65	6,363	33.8
>65	1,016	9.2
*Female*	7,158	51.5
*Married*	2,983	25.1
*Education*		
< High school	4,604	33.0
High School	3,006	27.6
Some college	1,474	12.3
≥ College	2,814	27.1
*Household income*		
Less than $30,000	7,636	60.8
$30,000 or more	3,647	32.5
*Has health insurance*	6,045	50.2
*Place of birth*		
US/Territory born	2,759	27.7
Foreign born in United States <10 years	7,253	51.2
Foreign born in United States ≥10 years	1,897	21.1
*Self-reported heritage*		
Central American/South American	1,227	7.4
Cuban	1,088	19.0
Dominican	1,582	10.2
Mexican	4,895	38.7
Puerto Rican	1,953	15.9
South American	821	5.12
More than one/other heritage	333	3.6
Spanish language preference	9,733	76.4
*Study site*		
the Bronx	2,941	24.7
Chicago	3,276	27.5
Miami	2,742	23.0
San Diego	2,950	24.8
*American Cancer Society Guideline adherence category* (1)		
Low	1,701	14.3
Moderate	7,125	59.8
High	3,083	25.9

Overall, Hispanic/Latino adults with high (weighted mean ± SE: −0.14 ± 0.06) or moderate (−0.03 ± 0.05) guideline adherence lived in neighborhoods with lower deprivation relative to adults with low guideline adherence (0.07 ± 0.07; *F statistic =* 19.77, *P* <0.001; Table 2). Moderate levels of income inequality were found for high (4.42 ± 0.03), moderate (4.36 ± 0.03), and low (4.35 ± 0.03; *F statistic = 8.94*, *P* <0.0001) guideline adherence, although the qualitative differences are minimal. Low levels of gentrification were observed for high (0.22 ± 0.13), moderate (0.09 ± 0.12), and low (0.12 ± 0.16; *F statistic* = 0.31, *P* = 0.734) guideline adherence groups.

**TABLE 2:** Weighted^a^ mean and SE for measures of change in neighborhood SES of U.S. Hispanic/Latino adults, by American Cancer Society Guideline Adherence

		American Cancer Society Guideline Adherence Categories^b^				
		Low	Moderate	High		
	No. of study participants	1,710	7,156	3,091	F statistic	*P*
Neighborhood deprivation index^c^	11,909	0.07 ± 0.07	−0.03 ± 0.05	−0.14 ± 0.06	19.77	<0.001
Neighborhood change in income inequality^d^	11,909	4.42 ± 0.03	4.36 ± 0.02	4.35 ± 0.03	8.94	<0.0001
Gentrification^e^	11,905	0.12 ± 0.15	0.09 ± 0.11	0.22 ± 0.13	0.31	0.734

Abbreviations: SE, standard error; SD, standard deviation.

^a^Analysis accounted for inverse probability weights for missing accelerometry data.

^b^Operationalization of the guideline adherence categories have been described in Table 1.

^c^Neighborhood deprivation index was calculated using 2010 census tract data, using principal component analysis with for six variables: percent of residents with less than a high school diploma, percent of residents with household incomes below 100% of the federal poverty level, percent of residents who are unemployed, and median household income. The score was standardized to have a mean of 0 and SD 1. Lower values of the index indicate lower deprivation and higher values indicate higher deprivation.

^d^Change in income inequality was measured using the 2005–2009 and the 2012–2016 Gini coefficient of income distribution to estimate the absolute percent change in income inequality percent change, scaled to 10. The Gini coefficient of income distribution can range from 0 (prefect equality) to 100 (perfect inequality), thus, a 1-unit change represents a 10% increase in inequality.

^e^Gentrification was operationalized using data from the 2000 decennial census and the 2006–10 American Community Survey, to calculate an index that captured percent change of college or more educated adults aged 25 or more, number of residents living below the federal poverty line, and median household income. Interpretation is based on a 1-unit change, with higher values indicate greater gentrification.

### Associations for Overall American Cancer Society Guideline Adherence

A 1-SD increase in the NDI was associated with a 13% decrease in the likelihood of high versus low guideline adherence (RRR = 0.87, 95% CI = 0.78–0.98), while no association was found for moderate adherence (RRR = 0.96, 95% CI = 0.86–1.06; Table 3). Adjusting for individual level SES attenuated the association for high guideline adherence (RRR = 0.89, 95% CI = 0.80–1.00). No associations were found between the Gini income inequality index or the gentrification index and overall guideline adherence score. Further adjustments for individual- or neighborhood-level covariates did not change these associations.

**TABLE 3:** Multinominal associations between measures of change in neighborhood SES of U.S. Hispanic/Latino adults and adherence to the 2012 American Cancer Society Guidelines on Nutrition and Physical Activity for Cancer Prevention

	American Cancer Society Guideline Adherence Categories^a^						
	Model 1, RRR (95% CI)^b^^,^^c^			Model 2, RRR (95% CI)^b^^,^^d^		Model 3, RRR (95% CI)^b^^,^^d^	
	Low	Moderate	High	Moderate	High	Moderate	High
Neighborhood deprivation index^e^	1.00	0.96 (0.86–1.06)	0.87 (0.78–0.98)	0.97 (0.87–1.07)	0.89 (0.80–1.00)	NA	NA
Neighborhood change in income inequality^d^	1.00	0.98 (0.80–1.20)	1.10 (0.89–1.37)	0.98 (0.80–1.20)	1.12 (0.89–1.40)	0.98 (0.80–1.21)	1.17 (0.93–1.48)
Gentrification^f^	1.00	0.99 (0.95–1.03)	1.02 (0.97–1.07)	0.99 (0.95–1.03)	1.02 (0.97–1.07)	0.98 (0.94–1.02)	1.01 (0.96–1.06)

Abbreviation: RRR, Relative Risk Ratio; SES, socioeconomic status; CI, Confidence Interval, SD, standard deviation.

^a^Operationalization of the guideline adherence categories is described in Table 1.

^b^Analysis accounted for inverse probability weights for missing accelerometry data.

^c^Model 1 adjusted for individual level covariates: age (18–44, 45–65, >65), sex (female, male), married (yes/no), health insurance status (insured/uninsured), combined nativity and years in the U.S. (foreign born and <10 years in U.S., foreign born and 10+ years in U.S., US born), language preference (Spanish, English), Heritage (Central or South American/more than 1 heritage/other, Cuban, Dominican, Mexican, Puerto Rican), study site (the Bronx, Chicago, Miami, San Diego).

^d^Model 2 additionally added individual-level socioeconomic status covariates: education (<high school, high school or GED, some college, college), household income (less than $30,000, $30,000 or more, missing).

^e^Model 3 added neighborhood-level covariates: percent foreign born (continuous) and neighborhood deprivation index.

^f^Operationalization of each neighborhood measure is described in Table 2. Neighborhood deprivation is interpreted as a 1-SD change with lower values of the index indicate lower deprivation and higher values indicate higher deprivation; Gini income inequality is interpreted as 10-unit change, thus, a 1-unit change represents a 10% increase in inequality; Gentrification index is interpreted as a 1-unit change, with higher scores reflecting greater gentrification.

### Exploratory Analysis with ACS Components

Table 4 provides estimates for the multinominal associations between neighborhood measures and individual guideline components. For recommendations regarding BMI, a one-SD increase in the NDI was associated with a 10% lower risk of being in the moderate adherence group (95% CI = 0.84–0.97) and 14% lower risk of being in the high adherence group (95% CI = 0.79–0.95) instead of the low adherence group. There was evidence of a statistically significant and positive association between gentrification and meeting the dietary guidelines moderately (low vs. moderate adherence RRR = 1.04, 95% CI = 1.01–1.07), but not high adherence (low vs. high adherence RRR = 1.02, 95% CI = 0.97–1.07). Gentrification was not associated with any other guideline components; while change in income inequality was not associated with any guideline components.

**TABLE 4:** Multinominal logistic regression for the associations between measures of change in neighborhood SES and individual components of the 2012 American Cancer Society Guidelines on Nutrition and Physical Activity for Cancer Prevention

	Neighborhood deprivation index^a^^,^^b^	Neighborhood change in income inequality^a^^,^^b^	Gentrification^a^^,^^b^
No. of study participants	11,909	11,909	11,905
	RRR (95% CI)		
American Cancer Society Guideline Components^c^^,^^d^			
Alcohol^c^^,^^d^			
Low	1.00	1.00	1.00
Moderate	0.88 (0.74–1.04)	0.98 (0.69–1.41)	1.00 (0.93–1.08)
High	0.99 (0.83–1.18)	1.16 (0.82–1.64)	0.96 (0.90–1.03)
Dietary^c^^,^^e^			
Low	1.00	1.00	1.00
Moderate	0.97 (0.90–1.04)	0.96 (0.84–1.10)	1.04 (1.01–1.07)
High	0.92 (0.79–1.07)	1.03 (0.75–1.41)	1.02 (0.97–1.07)
Body mass index^c^^,^^e^			
Low	1.00	1.00	1.00
Moderate	0.90 (0.84–0.97)	0.98 (0.85–1.12)	0.99 (0.96–1.02)
High	0.86 (0.79–0.95)	1.05 (0.88–1.26)	1.00 (0.96–1.04)
Body mass index^c^^,^^e^^,^^f^			
Low	1.00	1.00	1.00
Moderate	0.86 (0.78–0.95)	1.05 (0.88–1.26)	0.97 (0.93–1.01)
High	0.87 (0.77–0.97)	1.13 (0.91–1.41)	0.97 (0.92–1.03)
Physical activity^c^^,^^d^			
Low	1.00	1.00	1.00
Moderate	0.93 (0.73–1.19)	0.79 (0.50–1.23)	1.05 (0.97–1.13)
High	0.99 (0.76–1.30)	1.02 (0.64–1.62)	1.06 (0.98–1.15)

Abbreviation: RRR, Relative Risk Ratio; SES, socioeconimic status; CI, Confidence Interval; SD, standard deviation.

^a^Operationalization of each neighborhood measure is described in Table 2. Neighborhood deprivation is interpreted as a 1-SD change with lower values of the index indicate lower deprivation and higher values indicate higher deprivation; Gini income inequality is interpreted as 10-unit change, thus, a 1-unit change represents a 10% increase in inequality; Gentrification index is interpreted as a 1-unit change, with higher scores reflecting greater gentrification.

^b^All models adjusted for individual level covariates: age (18–44, 45–65, >65), sex (female, male), married (yes/no), health insurance status (insured/uninsured), combined nativity and years in the U.S. (foreign born and <10 years in U.S., foreign born and 10+ years in U.S., US born), language preference (Spanish, English), Heritage (Central or South American/more than 1 heritage/other, Cuban, Dominican, Mexican, Puerto Rican), study site (the Bronx, Chicago, Miami, San Diego), education (<high school, high school or GED, some college, college), household income (less than $30,000, $30,000 or more, missing). Gentrification and Gini income inequality models additionally adjusted for percent foreign born (continuous), while Gini income inequality models also added neighborhood deprivation index.

^c^Operationalization of the American Cancer Society guideline adherence categories is described in Table 1.

^d^Analysis accounted for inverse probability weights for missing accelerometry data.

^e^Analysis accounted for complex survey weights for study design.

^f^Sample restricted to never smokers.

### Exploratory Analysis Restricted to Never Smokers

Among 7,236 Hispanic/Latino adults who were never smokers (Table 5), a 1-unit increase in the NDI was associated with a 16%–18% decrease in high versus low overall guideline adherence (model 1 RRR = 0.82, 95% CI = 0.71–0.95; model 2 RRR = 0.84, 95% CI = 0.84, 95% CI = 0.72–0.97). Neighborhood gentrification and change in income inequality were not associated with overall guideline adherence among never smokers. Given the interactive effects between smoking and obesity (73), the BMI recommendations were further examined among never smokers (Table 4), which remained robust to this exclusion (low vs. moderate adherence, RRR = 0.86, 95% CI = 0.7800.95; low vs. high adherence, RRR = 0.87, 95% CI = 0.77–0.97).

**TABLE 5:** Multinominal associations between measures of change in neighborhood SES of U.S. Hispanic/Latino adults and adherence to the 2012 American Cancer Society Guidelines on Nutrition and Physical Activity for Cancer Prevention, Restricted to 7,236 Never Smokers

	ACS Guideline Adherence Categories^a^						
	Model 1, RRR (95% CI)^b^^,^^e^			Model 2, RRR (95% CI)^c^^,^^e^		Model 3, RRR (95% CI)^d^^,^^e^	
	Low	Moderate	High	Moderate	High	Moderate	High
Neighborhood deprivation index^f^	1.00	0.91 (0.80–1.03)	0.82 (0.71–0.95)	0.93 (0.82–1.05)	0.84 (0.72–0.97)	NA	NA
Neighborhood change in income inequality^f^	1.00	0.92 (0.70–1.21)	1.04 (0.78–1.39)	0.93 (0.70–1.22)	1.06 (0.80–1.42)	0.94 (0.71–1.25)	1.14 (0.84–1.53)
Gentrification ^f^	1.00	0.99 (0.94–1.03)	1.01 (0.95–1.08)	0.98 (0.94–1.03)	1.01 (0.95–1.08)	0.98 (0.94–1.03)	1.01 (0.95–1.08)

Abbreviation: RRR, Relative Risk Ratio; SES, socioeconimic status; CI, Confidence Interval; SD, standard deviation.

^a^Operationalization of the guideline adherence categories is described in Table 1.

^b^Model 1 adjusted for individual level covariates: age (18–44, 45–65, >65), sex (female, male), married (yes/no), health insurance status (insured/uninsured), combined nativity and years in the U.S. (foreign born and <10 years in U.S., foreign born and 10+ years in U.S., US born), language preference (Spanish, English), Heritage (Central or South American/more than 1 heritage/other, Cuban, Dominican, Mexican, Puerto Rican), study site (the Bronx, Chicago, Miami, San Diego).

^c^Model 2 additionally added individual-level socioeconomic status covariates: education (<high school, high school or GED, some college, college), household income (less than $30,000, $30,000 or more, missing).

^d^Model 3 added neighborhood-level covariates: percent foreign born (continuous) in all models and neighborhood deprivation index in the Gini income inequality models.

^e^Analysis accounted for inverse probability weights for missing accelerometry data.

^f^Operationalization of each neighborhood measure is described in Table 2. Neighborhood deprivation is interpreted as a 1-standard deviation change with lower values of the index indicate lower deprivation and higher values indicate higher deprivation; Gini income inequality is interpreted as 10-unit change, thus, a 1-unit change represents a 10% increase in inequality; Gentrification index is interpreted as a 1-unit change, with higher scores reflecting greater gentrification.

### Sensitivity Analysis Using Ordinal Logistic Regressions Modeling

Most analyses were confirmed in the ordinal logistic regression models with some notable differences (Supplementary Tables S1–S3). The magnitude of association between neighborhood deprivation index and overall guideline adherence was more attenuated, ranging from 6% to 7% lower likelihood of guideline adherence for each 1-unit increase in neighborhood deprivation (model 1 OR = 0.92, 95% CI = 0.99–0.98; model 2 OR = 0.93, 95% CI = 0.87–0.99, Supplementary Table S1). Findings were similar and of slightly stronger magnitude for analysis restricted to never smokers (Supplementary Table S2).

Weak to moderate correlations were observed between cross-sectional and longitudinal neighborhood SES indices with neighborhood deprivation and Gini income inequality being the highest, 0.38 (Supplementary Table S4).

## Discussion

In this large community-based study of 11,909 U.S. Hispanic/Latino adults representative of populations living in the Bronx, Chicago, Miami, and San Diego, we examined the roles of neighborhood socioeconomic change measures on adherence to healthful lifestyles in accordance with the American Cancer Society guidelines on nutrition, physical activity, and BMI for cancer prevention. In our stepwise multivariable adjusted analysis, we found evidence of an inverse association between neighborhood deprivation and guideline adherence. Hispanic/Latino adults residing in neighborhoods with increasing deprivation were 11%–18% less likely to have high overall guideline adherence relative to adults with low adherence. Of note, this association was attenuated with the inclusion of individual level SES in the model, suggesting that individual's baseline SES may serve as a buffer to their residential economic environment. We also found evidence of a negative association between neighborhood deprivation and individual guideline components: higher neighborhood deprivation associated with a 10%–14% lower likelihood to meet the BMI recommendations. Our findings corroborate prior work by several authors of this study has linked neighborhood deprivation (measured by residence level buffer) to BMI at baseline and changes in BMI over time among the San Diego subsample of Hispanic/Latino adults participating in the HCHS/SOL study (76).

Given their history of disinvestment, communities of color are at high risk of gentrification (77, 78) as cities now look to “revitalize” and “regenerate” these socioeconomically and racially segregated neighborhoods through new or improved developments in housing and commerce, creation of green spaces, and bringing in healthy food options like high-end grocery stores and farmers markets (42, 79). While long-time residents of these areas ought to benefit from improvements to their neighborhood, they are often excluded from the process, facing significant displacement to their businesses, political power, and social and cultural capital (38, 46, 47, 80). Long-time residents are also vulnerable to voluntary or involuntary physical displacement—and likely resegregation to environments that are worse off than their original place of residency (40, 81). Because of these vulnerabilities, it is hypothesized that gentrification may protect the health of more privileged residents rather than that of less privileged residents (40). In addition, gentrification may widen income gaps thereby exacerbating income inequality (38), with areas undergoing gentrification experiencing growing income inequality (82), which in turn is also linked to health inequities (83) and mortality (84, 85).

The literature on gentrification and change in income inequality and objective health measures remains scarce. Prior studies have linked increasing income inequality to worse health outcomes including adverse lifestyle behaviors (i.e., lower fruit and vegetable consumption; refs. 84–86). Contrary to this literature, we found that increasing income inequality did not associate with adherence to the overall guidelines or the individual guideline components. The null association in our study may be in part explained by the use of tract-level Gini index for income inequality which is distinct from all prior work that measured it at larger scales (i.e., state, counties, cities). Generally, studies have shown the gentrification is not associated with improved self-rated health (48), incidence of metabolic syndrome (39) or hypertension (50), and mortality (51) or may actually lead to worse outcomes (87) among minoritized residents relative to non-Hispanic White residents. Our findings are consistent with the hypothesis that gentrification may not directly benefit minoritized individuals in communities of color undergoing gentrification. Although retail and green gentrification may introduce high-quality physical activity resources (green spaces, recreational amenities) and supermarkets with higher quality foods (40), unaffordable rent and food prices may lead to food insecurity (88) among low-income residents and displacement for long-standing residents in the later stages of gentrification, especially in neighborhoods that undergo food gentrification (40, 88). Any benefits of gentrification in communities of color may only hold for residents of higher socioeconomic standing (89). For example, a study by Schnake-Mahl and colleagues among non-Hispanic Black, low-income mothers, found no association between displacement to a gentrified neighborhood with BMI and self-reported physical or mental health (49). Future studies that examine interactions between gentrification and individual SES are warranted. Previously, several authors of this study reported no association between gentrification and change in income inequality with 6-year incidence of metabolic syndrome among Hispanic/Latino adults (39). Consistent with these findings, in the current study, we found that gentrification or change in income inequality were not associated with the overall American Cancer Society guideline recommendations and individual guideline components like physical activity or alcohol intake. However, contrary to the existing literature, we found evidence suggesting a benefit of gentrification on dietary patterns. There are two notable factors that may contribute to this association. First, our measure of dietary adherence was based on operationalization of the 2012 guidelines (90) which did not incorporate operationalization of sugar-sweetened beverages unlike more recently updated guidelines (2) or other commonly used diet quality scales like the Healthy Eating Index (91). Future studies should examine this association further with other measures of diet quality and patterns. Second, it is important to note that HCHS/SOL participants may reside in neighborhoods that are at early stages of gentrification. Betancur and colleagues suggest that in the short term, gentrification may exert positive effects among Hispanic/Latino communities. However, in the long term, gentrification may have negative health effects via physical and sociocultural displacement and resource competition (92, 93). It is plausible that the full health effects of gentrification may be better captured over time through use of longitudinal or life course data or at a different geographical unit (e.g., block). In addition, the gentrification of Hispanic/Latino communities often occurs block to block with displacement manifesting at the block-level, resulting in residential mobility and resegregation due to unaffordable rent, increases in housing discrimination and ethno-racial profiling (94–96). This study lacked block-level census data. Furthermore, it is plausible that residents benefiting from gentrification may have higher socioeconomic standing, which this study was unable to disentangle due to insufficient power. Finally, given that income inequality is a process interrelated with gentrification, a nonsignificant relationship is in line with those of the gentrification models. Future work should examine types, stages, and other gentrification-related processes to better understand the role of gentrification as it develops over time on cancer-related disparities. This information may guide tailoring of cancer preventive interventions for at risk Hispanic/Latino communities.

Important strengths of this study include the use of probability sampling that enabled generalizability to the Hispanic/Latino target communities surrounding the study sites in the Bronx, Chicago, Miami, and San Diego compared with convenience samples (56). The use of validated dietary scales which considered cultural/traditional dietary patterns among Hispanic/Latino adults and objectively measured physical activity and BMI, captured behaviors more accurately. However, these measurements were taken at baseline, which prevent us from accounting for behavior changes over time and as guidelines have become more widely disseminated and awareness about them has increased. Our geocodes were coded to the census tract level and captured only at baseline. Therefore, we are unable to disentangle effects related to length of residence in a particular neighborhood and participant mobility (voluntary or involuntary) in and out of the baseline address (97). There is wide heterogeneity in how gentrification is measured (98) and our selected measurement may not fully or adequately capture the process of gentrification.

In conclusion, neighborhood deprivation, but not gentrification or changes in income inequality, was related to adherence to the 2012 American Cancer Society Guidelines on Nutrition and Physical Activity for Cancer Prevention. Our study suggests a need for community and population level interventions to increase uptake of healthful lifestyle behaviors to reduce cancer risk among the growing and aging U.S. Hispanic/Latino adult population residing in areas with high economic deprivation. Our study provides further evidence that gentrification or changes in income inequality of a neighborhood may not directly influence the health behaviors of Hispanic/Latino residents of these communities.

## Supplementary Material

eTable 1-4 eTable 1 - OLS estimates for nSES & ACS guidelines adherence
eTable 2 - OLS estimates for nSES & guidelines adherence among never smokers
eTable 3 - OLS estimates for nSES & guideline components
eTable 4- Correlations between neighborhood measures

## References

[1] Kushi LH , Doyle C, McCullough M, Rock CL, Demark-Wahnefried W, Bandera EV, . American Cancer Society Guidelines on nutrition and physical activity for cancer prevention: reducing the risk of cancer with healthy food choices and physical activity. CA Cancer J Clin 2012;62:30–67. 22237782 10.3322/caac.20140

[2] Rock CL , Thomson C, Gansler T, Gapstur SM, McCullough ML, Patel AV, . American Cancer Society guideline for diet and physical activity for cancer prevention. CA Cancer J Clin 2020;70:245–71. 32515498 10.3322/caac.21591

[3] Pichardo MS , Pichardo CM, Talavera GA, Gallo LC, Castañeda SF, Sotres-Alvarez D, . Neighborhood segregation and cancer prevention guideline adherence in US Hispanic/Latino adults: results from the HCHS/SOL. Front Oncol 2022;12:1024572. 36601483 10.3389/fonc.2022.1024572 PMC9806719

[4] Pichardo MS , Irwin ML, Esserman D, Molina Y, Ferrucci LM. A competing risk analysis of adherence to the American Cancer Society Guidelines on Nutrition and Physical Activity for Cancer Prevention and obesity-related cancer risk in Hispanic/Latino adults in the NIH-AARP Diet and Health Study. Int J Cancer 2022;151:1902–12. 35802472 10.1002/ijc.34200 PMC9588580

[5] Pichardo MS , Esserman D, Ferrucci LM, Molina Y, Chlebowski RT, Pan K, . Adherence to the American Cancer Society Guidelines on nutrition and physical activity for cancer prevention and obesity-related cancer risk and mortality in Black and Latina Women's Health Initiative participants. Cancer 2022;128:3630–40. 35996861 10.1002/cncr.34428

[6] Hales CM , Fryar CD, Carroll MD, Freedman DS, Ogden CL. Trends in obesity and severe obesity prevalence in US youth and adults by sex and age, 2007–2008 to 2015–2016. JAMA 2018;319:1723–5. 29570750 10.1001/jama.2018.3060 PMC5876828

[7] Reeves R , Rodrigue E, Kneebone E. Five evils: multidimensional poverty and race in America; 2016. Available from: https://www.brookings.edu/wp-content/uploads/2016/06/ReevesKneeboneRodrigue_MultidimensionalPoverty_FullPaper.pdf.

[8] Pichardo MS , Irwin ML, Sanft T, Ferruci LM, Ginader A, Nguyen TH, Esserman D, . A qualitative study identifying challenges resulting from complex evidence on lifestyle factors and cancer: perspectives from Black and Latina cancer survivors and health care providers. Support Care Cancer 2023;31:111 36633678 10.1007/s00520-022-07539-9 PMC9912693

[9] Gee GC , Ford CL. Structural racism and health inequities. Du Bois Rev 2011;8:115–32. 25632292 10.1017/S1742058X11000130 PMC4306458

[10] Marmot M . Society and the slow burn of inequality. Lancet 2020;395:1413–4. 32359458 10.1016/S0140-6736(20)30940-5 PMC7252106

[11] Laveist TA . Segregation, poverty, and empowerment: health consequences for African Americans. Milbank Q 1993;71:41–64. 8450822

[12] Coughlin SS . Social determinants of breast cancer risk, stage, and survival. Breast Cancer Res Treat 2019;177:537–48. 31270761 10.1007/s10549-019-05340-7

[13] Brulle RJ , Pellow DN. Environmental justice: human health and environmental inequalities. Annu Rev Public Health 2006;27:103–24. 16533111 10.1146/annurev.publhealth.27.021405.102124

[14] Taylor HL Jr , Hill W. Historical roots of the urban crisis: Blacks in the industrial city, 1900–1950. New York (NY): Routledge; 2013.

[15] Sangaramoorthy M , Yang J, Guan A, DeRouen MC, Tana MM, Somsouk M, . Asian American/Pacific Islander and Hispanic Ethnic Enclaves, Neighborhood Socioeconomic Status, and Hepatocellular Carcinoma Incidence in California: an update. Cancer Epidemiol Biomarkers Prev 2021;31:382–92. 34853019 10.1158/1055-9965.EPI-21-1035 PMC8825691

[16] Corral I , Landrine H, Zhao L. Residential segregation and obesity among a national sample of Hispanic adults. J Health Psychol 2014;19:503–8. 23460679 10.1177/1359105312474912

[17] Yu C-Y , Woo A, Hawkins C, Iman S. The impacts of residential segregation on obesity. J Phys Act Health 2018;15:834–9. 30314418 10.1123/jpah.2017-0352

[18] Hiatt RA , Breen N. The social determinants of cancer: a challenge for transdisciplinary science. Am J Prev Med 2008;35:S141–50. 18619394 10.1016/j.amepre.2008.05.006 PMC10773976

[19] Adler NE , Newman K. Socioeconomic disparities in health: pathways and policies. Health Aff 2002;21:60–76. 10.1377/hlthaff.21.2.60 11900187

[20] Mezuk B , Rafferty JA, Kershaw KN, Hudson D, Abdou CM, Lee H, . Reconsidering the role of social disadvantage in physical and mental health: stressful life events, health behaviors, race, and depression. Am J Epidemiol 2010;172:1238–49. 20884682 10.1093/aje/kwq283 PMC3025628

[21] Keller C , Fleury J. Factors related to physical activity in Hispanic women. J Cardiovasc Nurs 2006;21:142–5. 16601533 10.1097/00005082-200603000-00012

[22] Wen M , Maloney TN. Latino residential isolation and the risk of obesity in utah: the role of neighborhood socioeconomic, built-environmental, and subcultural context. J Immigr Minor Health 2011;13:1134–41. 21274631 10.1007/s10903-011-9439-8 PMC3132271

[23] Lee RE , Cubbin C. Neighborhood context and youth cardiovascular health behaviors. Am J Public Health 2002;92:428–36. 11867325 10.2105/ajph.92.3.428 PMC1447094

[24] Osypuk TL , Diez Roux AV, Hadley C, Kandula NR. Are immigrant enclaves healthy places to live? The Multi-ethnic Study of Atherosclerosis. Soc Sci Med 2009;69:110–20. 19427731 10.1016/j.socscimed.2009.04.010 PMC2750873

[25] Mellerson J , Landrine H, Hao Y, Corral I, Zhao L, Cooper DL. Residential segregation and exercise among a national sample of Hispanic adults. Health Place 2010;16:613–5. 20083422 10.1016/j.healthplace.2009.12.013

[26] Gouri Suresh SS , Schauder SA. Income segregation and access to healthy food. Am J Prev Med 2020;59:e31–8. 32418802 10.1016/j.amepre.2020.02.009

[27] Yi SS , Ruff RR, Jung M, Waddell EN. Racial/ethnic residential segregation, neighborhood poverty and urinary biomarkers of diet in New York City adults. Soc Sci Med 2014;122:122–9. 25441324 10.1016/j.socscimed.2014.10.030

[28] Ryabov I . Examining the role of residential segregation in explaining racial/ethnic gaps in spending on fruit and vegetables. Appetite 2016;98:74–9. 26721720 10.1016/j.appet.2015.12.024

[29] Havewala FD . The dynamics between the food environment and residential segregation: separate menus in metros, counties, and neighborhoods. Ph.D. The University of Texas at Dallas; 2016. Available from: https://search.proquest.com/docview/1802059700?accountid=15172; http://wa4py6yj8t.search.serialssolutions.com?ctx_ver=Z39.88–2004&ctx_enc=info:ofi/enc:UTF-8&rfr_id=info:sid/ProQuest+Dissertations+%26+Theses+Global&rft_val_fmt=info:ofi/fmt:kev:mtx:dissertation&rft.genre=dissertations+%26+theses&rft.jtitle=&rft.atitle=&rft.au=Havewala%2C+Ferzana+Dara&rft.aulast=Havewala&rft.aufirst=Ferzana&rft.date=2016–01–01&rft.volume=&rft.issue=&rft.spage=&rft.isbn=9781339768236&rft.btitle=&rft.title=The+dynamics+between+the+food+environment+and+residential+segregation%3A+Separate+menus+in+metros%2C+counties%2C+and+neighborhoods&rft.issn=&rft_id=info:doi/.

[30] Powell LM , Slater S, Chaloupka FJ, Harper D. Availability of physical activity-related facilities and neighborhood demographic and socioeconomic characteristics: a national study. Am J Public Health 2006;96:1676–80. 16873753 10.2105/AJPH.2005.065573 PMC1551946

[31] Cozier YC , Yu J, Coogan PF, Bethea TN, Rosenberg L, Palmer JR. Racism, segregation, and risk of obesity in the Black Women's Health Study. Am J Epidemiol 2014;179:875–83. 24585257 10.1093/aje/kwu004 PMC3969538

[32] Camacho-Rivera M , Rosenbaum E, Yama C, Chambers E. Low-income housing rental assistance, perceptions of neighborhood food environment, and dietary patterns among Latino adults: the AHOME study. J Racial Ethn Health Disparities 2017;4:346–53. 27129854 10.1007/s40615-016-0234-z PMC5685514

[33] Gao Y , Hickson DA, Talegawkar S, Norwood AF, Tucker KL, Sims M, . Influence of individual life course and neighbourhood socioeconomic position on dietary intake in African Americans: the Jackson Heart Study. BMJ Open 2019;9:e025237. 10.1136/bmjopen-2018-025237 PMC6429841 30862633

[34] Moore LV , Diez Roux AV. Associations of neighborhood characteristics with the location and type of food stores. Am J Public Health 2006;96:325–31. 16380567 10.2105/AJPH.2004.058040 PMC1470485

[35] Zenk SN , Schulz AJ, Israel BA, James SA, Bao S, Wilson ML. Neighborhood racial composition, neighborhood poverty, and the spatial accessibility of supermarkets in metropolitan Detroit. Am J Public Health 2005;95:660–7. 15798127 10.2105/AJPH.2004.042150 PMC1449238

[36] Child ST , Kaczynski AT, Fair ML, Stowe EW, Hughey SM, Boeckermann L, . ‘We need a safe, walkable way to connect our sisters and brothers’: a qualitative study of opportunities and challenges for neighborhood-based physical activity among residents of low-income African-American communities. Ethn Health 2019;24:353–64. 28707547 10.1080/13557858.2017.1351923

[37] Krieger N , Kim R, Feldman J, Waterman PD. Using the Index of Concentration at the Extremes at multiple geographical levels to monitor health inequities in an era of growing spatial social polarization: Massachusetts, USA (2010–14). Int J Epidemiol 2018;47:788–819. 29522187 10.1093/ije/dyy004

[38] Schnake-Mahl AS , Jahn JL, Subramanian SV, Waters MC, Arcaya M. Gentrification, neighborhood change, and population health: a systematic review. J Urban Health 2020;97:1–25. 31938975 10.1007/s11524-019-00400-1 PMC7010901

[39] Pichardo CM , Chambers EC, Sanchez-Johnsen LAP, Pichardo MS, Gallo LC, Talavera GA, . Association of census-tract level gentrification and income inequality with 6-year incidence of metabolic syndrome in the Hispanic Community Health Study/Study of Latinos, an epidemiologic cohort study. Soc Sci Med 2023:116222. 37776783 10.1016/j.socscimed.2023.116222 PMC11185427

[40] Cole HVS , Mehdipanah R, Gullon P, Triguero-Mas M. Breaking down and building up: gentrification, its drivers, and urban health inequality. Curr Environ Health Rep 2021;8:157–66. 33713334 10.1007/s40572-021-00309-5 PMC7955692

[41] Smith N . Gentrification. In: Van Vliet W, editor. The encyclopedia of housing. Thousand Oaks (CA): SAGE Publications; 1998.

[42] Rhodes-Bratton B , Rundle A, Lovasi GS, Herbstman J. The relationship between childhood obesity and neighborhood food ecology explored through the context of gentrification in New York City. Int Public Health J 2018;10:481–96. 32704343 PMC7377337

[43] Kershaw KN , Albrecht SS, Carnethon MR. Racial and ethnic residential segregation, the neighborhood socioeconomic environment, and obesity among Blacks and Mexican Americans. Am J Epidemiol 2013;177:299–309. 23337312 10.1093/aje/kws372 PMC3566709

[44] Gary-Webb TL , Egnot NS, Nugroho A, Dubowitz T, Troxel WM. Changes in perceptions of neighborhood environment and cardiometabolic outcomes in two predominantly African American neighborhoods. BMC Public Health 2020;20:52. 31937271 10.1186/s12889-019-8119-9 PMC6961335

[45] Anderson KF , Fullerton AS. Residential segregation, health, and health care: answering the Latino question. Race Soc Probl 2014;6:262–79.

[46] Atkinson R . The hidden costs of gentrification: displacement in central London. J Hous Built Environ 2000;15:307–26.

[47] Martin L . Fighting for control: political displacement in Atlanta's gentrifying neighborhoods. Urban Affairs Rev 2007;42:603–28.

[48] Gibbons J , Barton MS. The association of minority self-rated health with Black versus White gentrification. J Urban Health 2016;93:909–22. 27761683 10.1007/s11524-016-0087-0 PMC5126023

[49] Schnake-Mahl A , Sommers BD, Subramanian SV, Waters MC, Arcaya M. Effects of gentrification on health status after Hurricane Katrina. Health Place 2020;61:102237. 31740125 10.1016/j.healthplace.2019.102237 PMC7183421

[50] Morenoff JD , House JS, Hansen BB, Williams DR, Kaplan GA, Hunte HE. Understanding social disparities in hypertension prevalence, awareness, treatment, and control: the role of neighborhood context. Soc Sci Med 2007;65:1853–66. 17640788 10.1016/j.socscimed.2007.05.038 PMC2705439

[51] Huynh M , Maroko AR. Gentrification and preterm birth in New York City, 2008–2010. J Urban Health 2014;91:211–20. 24022181 10.1007/s11524-013-9823-x PMC3907632

[52] Chapple K . Income inequality and urban displacement: the new gentrification. Los Angeles (CA): SAGE Publications; 2017. p. 84–93.

[53] Walks RA , Maaranen R. Gentrification, social mix, and social polarization: testing the linkages in large Canadian cities. Urban Geography 2008;29:293–326.

[54] Smith BP , Madak-Erdogan Z. Urban neighborhood and residential factors associated with breast cancer in African American women: a systematic review. Horm Cancer 2018;9:71–81. 29417390 10.1007/s12672-018-0325-x PMC10355946

[55] Landrine H , Corral I, Lee JGL, Efird JT, Hall MB, Bess JJ. Residential segregation and racial cancer disparities: a systematic review. J Racial Ethn Health Disparities 2017;4:1195–205. 28039602 10.1007/s40615-016-0326-9

[56] LaVange LM , Kalsbeek WD, Sorlie PD, Avilés-Santa LM, Kaplan RC, Barnhart J, . Sample design and cohort selection in the Hispanic community health study/study of Latinos. Ann Epidemiol 2010;20:642–9. 20609344 10.1016/j.annepidem.2010.05.006 PMC2921622

[57] Sorlie PD , Avilés-Santa LM, Wassertheil-Smoller S, Kaplan RC, Daviglus ML, Giachello AL, . Design and implementation of the Hispanic community health study/study of Latinos. Ann Epidemiol 2010;20:629–41. 20609343 10.1016/j.annepidem.2010.03.015 PMC2904957

[58] Manson S , Schroeder J, Van Riper D, Ruggles S. Data from: IPUMS National Historical Geographic Information System: Version 14.0 0 [database]; 2019.

[59] Geolytics. Data from: Neighborhood change database [NCDB] tract data from 1970–2010 [online demographic data]; 2014.

[60] Messer LC , Laraia BA, Kaufman JS, Eyster J, Holzman C, Culhane J, . The development of a standardized neighborhood deprivation index. J Urban Health 2006;83:1041–62. 17031568 10.1007/s11524-006-9094-x PMC3261293

[61] Pichardo CM , Pichardo MS, Gallo LC, Talavera GA, Chambers EC, Sanchez-Johnsen LAP, . Association of neighborhood segregation with 6-year incidence of metabolic syndrome in the Hispanic community health study/study of Latinos. Ann Epidemiol 2023;78:1–8. 36473628 10.1016/j.annepidem.2022.11.003 PMC10127516

[62] US Census Bureau. Shrider EA, Kollar M, Chen F, Semega J. Income and poverty in the United States: 2020. Current Population Reports. p. 60–273.

[63] Iceland J , Weinberg DH, Steinmetz E. Racial and ethnic residential segregation in the United States 1980–2000. Vol 8. No. 3. Bureau of Census; 2002.

[64] Cubbin C , Kim Y, Vohra-Gupta S, Margerison C. Longitudinal measures of neighborhood poverty and income inequality are associated with adverse birth outcomes in Texas. Soc Sci Med 2020;245:112665. 31778899 10.1016/j.socscimed.2019.112665 PMC8601022

[65] Linton SL , Cooper HLF, Kelley ME, Karnes CC, Ross Z, Wolfe ME, . Cross-sectional association between ZIP code-level gentrification and homelessness among a large community-based sample of people who inject drugs in 19 US cities. BMJ Open 2017;7:e013823. 10.1136/bmjopen-2016-013823 PMC5541298 28637724

[66] Siega-Riz AM , Sotres-Alvarez D, Ayala GX, Ginsberg M, Himes JH, Liu K, . Food-group and nutrient-density intakes by Hispanic and Latino backgrounds in the Hispanic Community Health Study/Study of Latinos. Am J Clin Nutr 2014;99:1487–98. 24760972 10.3945/ajcn.113.082685 PMC4021787

[67] Daviglus ML , Talavera GA, Avilés-Santa ML, Allison M, Cai J, Criqui MH, . Prevalence of major cardiovascular risk factors and cardiovascular diseases among Hispanic/Latino individuals of diverse backgrounds in the United States. JAMA 2012;308:1775–84. 23117778 10.1001/jama.2012.14517 PMC3777250

[68] Thomson CA , McCullough ML, Wertheim BC, Chlebowski RT, Martinez ME, Stefanick ML, . Nutrition and physical activity cancer prevention guidelines, cancer risk, and mortality in the Women's Health Initiative. Cancer Prev Res 2014;7:42–53. 10.1158/1940-6207.CAPR-13-0258 PMC4090781 24403289

[69] Castaneda SF , Garcia ML, Lopez-Gurrola M, Stoutenberg M, Emory K, Daviglus ML, . Alcohol use, acculturation and socioeconomic status among Hispanic/Latino men and women: The Hispanic Community Health Study/Study of Latinos. PLoS One 2019;14:e0214906. 30947280 10.1371/journal.pone.0214906 PMC6449031

[70] Evenson KR , Sotres-Alvarez D, Deng YU, Marshall SJ, Isasi CR, Esliger DW, . Accelerometer adherence and performance in a cohort study of US Hispanic adults. Med Sci Sports Exerc 2015;47:725–34. 25137369 10.1249/MSS.0000000000000478 PMC4333140

[71] Gallo LC , Penedo FJ, Carnethon M, Isasi CR, Sotres-Alvarez D, Malcarne VL, . The Hispanic Community Health Study/Study of Latinos Sociocultural Ancillary Study: sample, design, and procedures. Ethn Dis 2014;24:77–83. 24620452 PMC3986116

[72] Seaman SR , White IR. Review of inverse probability weighting for dealing with missing data. Stat Methods Med Res 2011;22:278–95. 21220355 10.1177/0962280210395740

[73] Song M , Giovannucci E. Estimating the influence of obesity on cancer risk: stratification by smoking is critical. J Clin Oncol 2016;34:3237–9. 27458311 10.1200/JCO.2016.67.6916

[74] Agresti A . Chapter 5. Building and applying logistic regression models. An introduction to categorical data analysis. Hoboken (NJ): John Wiley & Sons; 2018.

[75] Stata/SE 16.1 for Mac (Intel 64-bit). 1985–2019 StataCorp LLC; 2021. Available from: http://www.stata.com.

[76] Gallo LC , Savin KL, Jankowska MM, Roesch SC, Sallis JF, Sotres-Alvarez D, . Neighborhood environment and metabolic risk in Hispanics/Latinos from the Hispanic community health study/study of latinos. Am J Prev Med 2022;63:195–203. 35365395 10.1016/j.amepre.2022.01.025 PMC9308627

[77] DeVerteuil G . Immigration and gentrification. Handbook of gentrification studies. London: Edward Elgar Publishing; 2018. p. 428–43.

[78] Hwang J . Gentrification in changing cities. Ann Am Acad Pol Soc Sci 2015;660:319–40.

[79] Smith N . Gentrification and uneven development. Econ Geogr 1982;58:20–8.12265156

[80] Sullivan DM , Shaw SC. Retail gentrification and race: the case of Alberta Street in Portland, Oregon. Urban Affairs Rev 2011;47:413–32.

[81] Mehdipanah R , Manzano A, Borrell C, Malmusi D, Rodriguez-Sanz M, Greenhalgh J, . Exploring complex causal pathways between urban renewal, health and health inequality using a theory-driven realist approach. Soc Sci Med 2015;124:266–74. 25486624 10.1016/j.socscimed.2014.11.050

[82] Christafore D , Leguizamon S. Neighbourhood inequality spillover effects of gentrification. Pap Reg Sci 2018;98:1469–84.

[83] Barrett RE , Cho YI, Weaver KE, Ryu K, Campbell RT, Dolecek TA, . Neighborhood change and distant metastasis at diagnosis of breast cancer. Ann Epidemiol 2008;18:43–7. 17890103 10.1016/j.annepidem.2007.07.001

[84] Pickett KE , Wilkinson RG. Income inequality and health: a causal review. Soc Sci Med 2015;128:316–26. 25577953 10.1016/j.socscimed.2014.12.031

[85] Bor J , Cohen GH, Galea S. Population health in an era of rising income inequality: USA, 1980–2015. Lancet 2017;389:1475–90. 28402829 10.1016/S0140-6736(17)30571-8

[86] Horino M , Liu SY, Lee EY, Kawachi I, Pabayo R. State-level income inequality and the odds for meeting fruit and vegetable recommendations among US adults. PLoS One 2020;15:e0238577. 32903265 10.1371/journal.pone.0238577 PMC7480846

[87] Francis JM , Bhavsar NA, Kumar M, Richman L. Defining gentrification for epidemiologic research: a systematic review. PLoS One 2020;15:e0233361. 32437388 10.1371/journal.pone.0233361 PMC7241805

[88] Whittle HJ , Palar K, Hufstedler LL, Seligman HK, Frongillo EA, Weiser SD. Food insecurity, chronic illness, and gentrification in the San Francisco Bay Area: An example of structural violence in United States public policy. Soc Sci Med 2015;143:154–61. 26356827 10.1016/j.socscimed.2015.08.027

[89] Bhavsar NA , Kumar M, Richman L. Defining gentrification for epidemiologic research: a systematic review. PLoS One 2020;15:e0233361. 32437388 10.1371/journal.pone.0233361 PMC7241805

[90] Rock CL , Doyle C, Demark-Wahnefried W, Meyerhardt J, Courneya KS, Schwartz AL, . Nutrition and physical activity guidelines for cancer survivors. CA Cancer J Clin 2012;62:243–74. 22539238 10.3322/caac.21142

[91] Krebs-Smith SM , Pannucci TE, Subar AF, Kirkpatrick SI, Lerman JL, Tooze JA, . Update of the healthy eating index: HEI-2015. J Acad Nutr Diet 2018;118:1591–602. 30146071 10.1016/j.jand.2018.05.021 PMC6719291

[92] García I , Rúa MM. ‘Our interests matter’: Puerto Rican older adults in the age of gentrification. Urban Stud 2017;55:3168–84.

[93] Curran W . ‘Mexicans love red’ and other gentrification myths: displacements and contestations in the gentrification of Pilsen, Chicago, USA. Urban Stud 2018;55:1711–28.

[94] Betancur J . Gentrification and community fabric in Chicago. Urban Stud 2011;48:383–406. 21275200 10.1177/0042098009360680

[95] Fullilove MT . Psychiatric implications of displacement: contributions from the psychology of place. Am J Psychiatry 1996;153:1516–23. 8942445 10.1176/ajp.153.12.1516

[96] Byrne JP . Two cheers for gentrification. Howard Law J 2002;46:405–32.

[97] Perreira KM , Abreu MLA, Zhao B, Youngblood ME, Alvarado C, Cobo N, . Retaining hispanics: lessons from the Hispanic community health study/study of Latinos. Am J Epidemiol 2020;189:518–31. 31971236 10.1093/aje/kwaa003 PMC7523586

[98] Williams K . Toward a universal operationalization of gentrification. Sociation Today 2015;13:1.

