# Supplementary material for: Change in Neighborhood Socioeconomic Status and Adherence to the Cancer Prevention Lifestyle Guidelines in Hispanic/Latino Adults: Results from the HCHS/SOL Study
Source: Cancer Res Commun. 2023 Oct 2;3(10):1981–91. doi: 10.1158/2767-9764.CRC-23-0187 (PMC10542571; doi:10.1158/2767-9764.CRC-23-0187)
Supplement: eTable 1-4 — eTable 1 - OLS estimates for nSES & ACS guidelines adherence eTable 2 - OLS estimates for nSES & guidelines adherence among never smokers eTable 3 - OLS estimates for nSES & guideline components eTable 4- Correlations between neighborhood measures [file crc-23-0187-s01.docx]

**Supplementary Tables**

**Change in Neighborhood Socioeconomic Status and Adherence to the Cancer Prevention Lifestyle Guidelines in Hispanic/Latino Adults: Results from the HCHS/SOL Study**

**Authors**

Margaret S. Pichardo^1*^, Catherine M. Pichardo^2^, Gregory A. Talavera^3^, Linda C. Gallo^3^, Charlene C. Kuo^4^ ; Sheila F. Castañeda^3^, Earle C. Chambers^5^, Martha L. Daviglus^6^, Amber Pirzada^6^, Krista M. Perreira^7^, Daniela Sotres-Alvarez^8^, Tania Yadhira Pena Ortiz^9^, Jesse J. Plascak^10^

**Affiliations**

1 Department of Surgery, Hospital of the University of Pennsylvania, University of Pennsylvania Health System, Philadelphia, PA, USA.

2 South Bay Latino Research Center, Department of Psychology, San Diego State University, California, USA

3 Department of Psychology, San Diego State University, California, USA

4 Department of Behavioral and Community Health, University of Maryland School of Public Health, College Park, USA

5 Department of Family and Social Medicine, Albert Einstein College of Medicine, the Bronx, New York, USA

6 Institute for Minority Health Research, University of Illinois Chicago, Chicago, USA

7 Department of Social Medicine University of North Carolina School of Medicine, Chapel Hill, USA

8 Department of Biostatistics, University of North Carolina School of Medicine, Chapel Hill, USA

9 Weill Cornell Medical College, Weill Cornel Medicine, New York City, New York, USA

10 Division of Cancer Prevention and Control, Ohio State University College of Medicine, Columbus, USA

**Table of Content**

eTable 1. Ordered Logistic Regression Estimates for the Associations between Measures of Change in Neighborhood Socioeconomic Status of U.S. Hispanic/Latino Adults and Adherence to the 2012 ACS Nutrition and Physical Activity Guidelines for Cancer Prevention^1^

eTable 2. Ordered Logistic Regression Estimates for the Associations between Measures of Change in Neighborhood Socioeconomic Status and 2012 ACS Nutrition and Physical Activity Guidelines for Cancer Prevention^1^, Restricted to Never Smokers

eTable 3. Ordered Logistic Regression Estimates for the Associations between Measures of Change in Neighborhood Socioeconomic Status and Adherence to the 2012 ACS Nutrition and Physical Activity Guidelines for Cancer Prevention

eTable 4. Correlations between Measures of Neighborhood Socioeconomic Status

| eTable 1. Ordered Logistic Regression Estimates for the Associations between Measures of Change in Neighborhood Socioeconomic Status of U.S. Hispanic/Latino Adults and Adherence to the 2012 ACS Nutrition and Physical Activity Guidelines for Cancer Prevention^1^ | | | | |
| --- | --- | --- | --- | --- |
|  |  | Model 1^2,5^ | Model 2^3,5^ | Model 3^4,5^ |
|  | No. of study participants | OR (95% CI) |  |  |
| Neighborhood deprivation index^6^ | 11,909 | 0.92 (0.86, 0.98) | 0.93 (0.87, 0.99) | NA |
| Neighborhood change in income inequality^6^ | 11,909 | 1.07 (0.94, 1.22) | 1.08 (0.95, 1.24) | 1.12 (0.98, 1.29) |
| Gentrification^6^ | 11,905 | 1.02 (0.99 1.05) | 1.02 (0.99, 1.05) | 1.02 (0.99, 1.05) |
| *Notes*. Abbreviations: ACS, American Cancer Society.  ^1^ Operationalization of the ACS guideline adherence categories is described in Table 1.  ^2^ Model 1 adjusted for individual level covariates: age (18-44, 45-65, >65), sex (female, male), married (yes/no), health insurance status (insured/uninsured), combined nativity and years in the U.S. (foreign born and <10 years in U.S., foreign born and 10+ years in U.S., US born), language preference (Spanish, English), Heritage (Central or South American/more than 1 heritage/other, Cuban, Dominican, Mexican, Puerto Rican), study site (the Bronx, Chicago, Miami, San Diego).  ^3^ Model 2 additionally added individual-level socioeconomic status covariates: education (<high school, high school or GED, some college, college), household income (less than $30,000, $30,000 or more, missing).  ^4^ Model 3 added neighborhood-level covariates: percent foreign born (continuous) in all models and neighborhood deprivation index in the GINI income inequality models.  ^5^ Analysis accounted for inverse probability weights for missing accelerometry data.  ^6^ Operationalization of each neighborhood measure is described in Table 2. Neighborhood deprivation is interpreted as a 1-standard deviation change; GINI income inequality is interpreted as 10-unit change, thus, a 1-unit change represents a 10% increase in inequality; Gentrification index is interpreted as a 1-unit change, with higher scores reflecting greater gentrification. | | | | |

| eTable 2. Ordered Logistic Regression Estimates for the Associations between Measures of Change in Neighborhood Socioeconomic Status and 2012 ACS Nutrition and Physical Activity Guidelines for Cancer Prevention^1^, Restricted to Never Smokers | | | | |
| --- | --- | --- | --- | --- |
|  |  | Model 1^2,5^ | Model 2^3,5^ | Model 3^4,5^ |
|  | No. of study participants | OR (95% CI) | | |
| Neighborhood deprivation index^6^ | 7,328 | 0.90 (0.82, 0.98) | 0.91 (0.83, 0.99) | NA |
| Neighborhood change in income inequality^6^ | 7,328 | 1.04 (0.89, 1.22) | 1.05 (0.90, 1.24) | 1.10 (0.94, 1.29) |
| Gentrification^6^ | 7,326 | 1.01 (0.98, 1.05) | 1.01 (0.98, 1.05) | 1.01 (0.98, 1.05) |
| *Notes*. Abbreviations: ACS, American Cancer Society.  ^1^ Operationalization of the ACS guideline adherence categories is described in Table 1.  ^2^ Model 1 adjusted for individual level covariates: age (18-44, 45-65, >65), sex (female, male), married (yes/no), health insurance status (insured/uninsured), combined nativity and years in the U.S. (foreign born and <10 years in U.S., foreign born and 10+ years in U.S., US born), language preference (Spanish, English), Heritage (Central or South American/more than 1 heritage/other, Cuban, Dominican, Mexican, Puerto Rican), study site (the Bronx, Chicago, Miami, San Diego).  ^3^ Model 2 additionally added individual-level socioeconomic status covariates: education (<high school, high school or GED, some college, college), household income (less than $30,000, $30,000 or more, missing).  ^4^ Model 3 added neighborhood-level covariates: percent foreign born (continuous) in all models and neighborhood deprivation index in the GINI income inequality models.  ^5^ Analysis accounted for inverse probability weights for missing accelerometry data.  ^6^ Operationalization of each neighborhood measure is described in Table 2. Neighborhood deprivation is interpreted as a 1-standard deviation change; GINI income inequality is interpreted as 10-unit change, thus, a 1-unit change represents a 10% increase in inequality; Gentrification index is interpreted as a 1-unit change, with higher scores reflecting greater gentrification. | | | | |

| eTable 3. Ordered Logistic Regression Estimates for the Associations between Measures of Change in Neighborhood Socioeconomic Status and Adherence to the Components of the 2012 ACS Nutrition and Physical Activity Guidelines for Cancer Prevention^1^ | | | |
| --- | --- | --- | --- |
|  | Neighborhood deprivation index^2,3^ | Neighborhood change in income inequality^2,4^ | Gentrification^2,5^ |
| No. of study participants | 11,909 | 11,909 | 11,905 |
|  | RRR (95% CI) | | |
| Alcohol^1,6^ | 1.08 (0.96, 1.21) | 1.17 (0.97, 1.41) | 0.96 (0.92, 1.01) |
|  |  |  |  |
| Dietary^1,6^ | 0.96 (0.90, 1.02) | 0.98 (0.86, 1.11) | 1.03 (1.00, 1.05) |
|  |  |  |  |
| Body Mass Index^1,6^ | 0.91 (0.85, 0.97) | 1.04 (0.92, 1.17) | 1.00 (0.97, 1.02) |
|  |  |  |  |
| Body Mass Index among never smokers (n=)^1,6,7^ | 0.90 (0.84, 0.98) | 1.09 (0.94, 1.26) | 0.98 (0.95, 1.01) |
|  |  |  |  |
| Physical Activity^1,8^ | 1.06 (0.97, 1.16) | 1.23 (1.06, 1.43) | 1.02 (0.99, 1.05) |
| *Notes*. Abbreviations: ACS, American Cancer Society.  ^1^ Operationalization of the ACS guideline adherence categories is described in Table 1.  ^2^ Operationalization of each neighborhood measure is described in Table 2. Neighborhood deprivation is interpreted as a 1-standard deviation change; GINI income inequality is interpreted as 10-unit change, thus, a 1-unit change represents a 10% increase in inequality; Gentrification index is interpreted as a 1-unit change, with higher scores reflecting greater gentrification. ^3^ All models adjusted for individual level covariates: age (18-44, 45-65, >65), sex (female, male), married (yes/no), health insurance status (insured/uninsured), combined nativity and years in the U.S. (foreign born and <10 years in U.S., foreign born and 10+ years in U.S., US born), language preference (Spanish, English), Heritage (Central or South American/more than 1 heritage/other, Cuban, Dominican, Mexican, Puerto Rican), study site (the Bronx, Chicago, Miami, San Diego), education (<high school, high school or GED, some college, college), household income (less than $30,000, $30,000 or more, missing).  ^4^ GINI income inequality model additionally adjusted for percent foreign born (continuous) and neighborhood deprivation index.  ^5^ Gentrification models additionally adjusted for percent foreign born (continuous).  ^6^ Analysis accounted for complex survey weights for study design.  ^7^ Analysis was restricted to never smokers.  ^8^Analysis accounted for inverse probability weights for missing accelerometry data. | | | |

| eTable 4. Correlations between Measures of Neighborhood Socioeconomic Status | | | | |
| --- | --- | --- | --- | --- |
|  | (1) | (2) | (3) | (4) |
| (1) Neighborhood deprivation index | 1 |  |  |  |
| (2) % Foreign Born | 0.0211 |  |  |  |
| (3) Neighborhood change in income inequality | 0.3800 | 0.1417 |  |  |
| (4) Gentrification | -0.1385 | -0.0888 | 0.1910 | 1 |
